# Okra (*Abelmoscus esculentus*) Improved Islets Structure, and Down-Regulated *PPARs* Gene Expression in Pancreas of High-Fat Diet and Streptozotocin-Induced Diabetic Rats

**DOI:** 10.22074/cellj.2018.4819

**Published:** 2018-01-01

**Authors:** Naeem Erfani Majd, Mohammad Reza Tabandeh, Ali Shahriari, Zahra Soleimani

**Affiliations:** 1 Department of Basic Sciences, Histology Section, Faculty of Veterinary Medicine, Shahid Chamran University of Ahvaz, Ahvaz, Iran; 2 Stem Cell and Transgenic Technology Research Center of Shahid Chamran University of Ahvaz, Ahvaz, Iran; 3 Department of Biochemistry and Molecular Biology Section, Faculty of Veterinary Medicine, Shahid Chamran University of Ahvaz, Ahvaz, Iran

**Keywords:** Diabetes, Okra, Pancreas, *PPARs*, Rat

## Abstract

**Objective:**

Okra (*Abelmoschus esculentus*) is a tropical vegetable that is rich in carbohydrates, fibers, proteins and natural antioxidants. The aim of the present study was to evaluate the effects of Okra powder on pancreatic islets and its action on the expression of *PPAR-γ* and *PPAR-α* genes in pancreas of high-fat diet (HFD) and streptozotocin- induced diabetic rats.

**Materials and Methods:**

In this experimental study, diabetes was induced by feeding HFD (60% fat) for 30 days followed by an injection of streptozotocin (STZ, 35 mg/kg). Okra powder (200 mg/kg) was given orally for 30 days after diabetes induction. At the end of the experiment, pancreas tissues were removed and stained by haematoxylin and Eozine and aldehyde fuchsin for determination of the number of β-cells in pancreatic islets. Fasting blood sugar (FBS), Triglycerides (TG), cholesterol, high density lipoprotein (HDL), low density lipoprotein (LDL), and insulin levels were measured in serum. Moreover, *PPAR-γ* and *PPAR-α* mRNAs expression were measured in pancreas using real time polymerase chain reaction (PCR) analysis.

**Results:**

Okra supplementation significantly decreased the elevated levels of FBS, total cholesterol, and TG and attenuated homeostasis model assessment of basal insulin resistance (HOMA-IR) index in diabetic rats. The expression levels of *PPAR-γ* and *PPAR-α* genes that were elevated in diabetic rats, attenuated in okra-treated rats (P<0.05). Furthermore, okra improved the histological damages of pancreas including vacuolization and decreased β-cells mass, in diabetic rats.

**Conclusion:**

Our findings confirmed the potential anti-hyperglycemic and hypolipidemic effects of Okra. These changes were associated with reduced pancreatic tissue damage. Down-regulation of *PPARs* genes in the pancreas of diabetic rats after treatment with okra, demonstrates that okra may improve glucose homeostasis and β-cells impairment in diabetes through a PPAR-dependent mechanism.

## Introduction

Type 2 diabetes (Fig T2D) and obesity are the most frequent endocrine-metabolic diseases that are characterized by hyperglycemia and impaired insulin action and secretion (i.e. insulin resistance) (1). Increasing evidence from epidemiological studies indicates that genetic predisposition and environmental factors including obesity and sedentary life style are major risk factors for the development of diabetes. Consumption of high amounts of prepared foods rich in sugar and fat increases the risk of dyslipidaemia, obesity, insulin resistance and diabetes (2). Most of the individuals diagnosed with T2D are found to be obese. Similarly, consumption of a high- fat diet (HFD) increases the risk of acute insulin resistance in rodents (3). 

Obesity is accompanied by various metabolic complications including dyslipidemia, hyperglycemia and increased levels of circulating cytokines. Both dyslipidemia and hyperglysemia contribute to loss of ß-cells function and impairment of insulin secretion in obesity and diabetes. Fatty acids (FAs) have been shown to be pro-apoptotic for ß-cells. Proliferative capacity of ß-cells is inhibited after a prolonged exposure to increased glucose concentrations. Considering the role of ß-cells pathophysiology in progression of T2D, genetic background seems to be crucially important and recent studies have shown that ß-cells dysfunction in hyperglycemic state is associated with down/up regulation of key islets genes (4). 

Peroxisome proliferator-activated receptors (PPARα, PPARγ and PPARβ/δ) are the members of the nuclear receptor superfamily which play crucial roles in regulating lipid and glucose homeostasis and controlling cellular proliferation in pancreatic endocrine tissue. The function of PPAR-γ on insulin sensitivity is due to its ability to channel FAs into adipose tissue; therefore, diminishing plasma FAs concentration and reducing lipotoxicity in the pancreas (5). Also *PPAR-γ* regulates insulin release from the pancreatic ß-cells by activating glucokinase and glucose transporter (GLUT2). Also, *PPAR-α* is expressed in rat pancreatic islets and severe activation of *PPAR-α* induces mitochondrial ß oxidation of FA and potentiates glucose- stimulated insulin secretion (GSIS) in rat islets (6). Therefore, *PPAR-α* agonists improve pancreatic ß-cells function in insulin-resistant rodents (7) and manipulation of *PPAR* signaling pathway is one of the most attractive approaches in drug discovery for the treatment of diabetes. 

In recent years, the use of different medicinal plants for the treatment of diabetes mellitus has increased. Medicinal herbs could be extensively used for the treatment of diabetic patients due to beneficial actions of their bioactive compounds on ß-cells function, as well as insulin action, production and resistance. Although, herbal medicines are considered for the treatment of diabetes mellitus, the efficacy of these medicinal plants and their derivatives in regulation of metabolic disorders is not completely investigated and many of them remain untested.

*Abelmoschus esculentus* belonging to Malvaceae family, is a plant native to Africa and India and has been a part of the diet in various parts of the world (8). Phytochemical studies exhibited that polysaccharides, polyphenols, flavonoids, tannins, sterols and triterpenes are the major components of *A. esculentus* with various biological activities (9). It has been reported that the okra powder plays antidiabetic and antihyperlipidemic roles in diabetic rats. Dietary fibers and polyphenols which are abundantly found in *A. esculentus*, may contribute to the hypoglycemic and hypolipidemic effects of *A. esculentus* as suggested previously (10). 

In spite of beneficial effects of *A. esculentus* for treating metabolic complications of diabetic patients, its impact on pancreatic histological and molecular changes associated with HFD-induced diabetes has not been clarified. This study was conducted to evaluate the effects of *A. esculentus* (okra) on histological change and *PPAR-γ* and *PPAR-α* gene expression in pancreas of HFD/ streptozotocin (STZ)-induced diabetic animal model.

## Materials and Methods

*A. esculentus* (a native cultivar of Khuzestan, Iran) was collected from local farms in Ahvaz, South-West of Iran. The plants were kindly identified by Plant Taxonomy laboratory, Faculty of Agriculture Science, Shahid Chamran University of Ahvaz, Ahvaz, Iran. The fruit (seed and peel) were washed with water and shade-dried at room temperature. The dried materials were grounded into fine powder using a mixer grinder. Then, the powder was weighed and kept away from moisture in plastic vials in desiccator for further use. Moisture of dried fruits was calculated based on the following formula: % moisture (w/w)=[(W_spl_W_dry_)/W_spl_]×100, where W_spl_ was the weight of sample before drying and W_dry_ was the weight of dried material. Total protein and sugar contents were determined using Bradford and PhenolSulfuric acid methods, respectively and reported as g/100 g dry material. 

### Measurement of flavonoids content

The flavonoids content (FC) was determined using the method suggested by Huang et al. (11) with minor modifications. For this purpose, 5 mL of 2% aluminium trichloride (AlCl3) in methanol was mixed with the same volume of *A. esculentus* powder (0.4 mg/ mL). Absorption of the resulting solution was read at 367 nm using UV- visible spectrophotometer (BioTek, CA, USA) against a blank sample containing 5 mL extract mixture dissolved in 5 mL methanol without AlCl3. FC was determined using a standard curve plotted using quercetin (0.2-1 mg/ml) as the standard and expressed as mg of quercetin equivalents per 100 g dry extract. All experiments were performed in triplicate.

### Measurement of phenolic content

Phenolic content (PC) of the samples were measured using Folin-Ciocalteu colorimetric method (12) with slight modifications. Here, 100 µL of *A. esculentus* powder was mixed with 0.5 mL Folin-Ciocalteu reagent (10 times diluted with distilled water). Next, 7 mL of distilled water was added to the mixture and it was left at room temperature for 5 minutes. Then, 1.5 mL sodium bicarbonate solution (60 mg/ ml) was added to the mixture and left at room temperature in the dark for 2 hours. Absorbance was read at 725 nm against blank using UV-visible spectrophotometer (BioTek, CA, USA). A calibration curve was constructed using a standard solution of gallic acid (0.2-1 mg/ml). Results were expressed as mg gallic acid per 100 g dry extract. All experiments were performed in triplicate.

### Animals and diets

In this experimental study, healthy adult female Wistar rats (200-220 g) were obtained from the experimental animal holding of Joundishapour University of Medical Sciences, Ahvaz, Iran. The animals were housed in standard cages and maintained at controlled room temperature (23 ± 1°C), with a relative humidity of 60 ± 5%, and 12 hour/12 hour light/darkcycles. They had ad libitum access to rat chow (Pars, Iran) and water. All animals were used according to the guidelines for the care and use of laboratory animals provided by thenational academy of sciences (National Institutes of Healthpublication No. 86-23). Approval for animal studies was obtained from the animal Ethics Committee of Faculty ofVeterinary Medicine, Shahid Chamran University of Ahvaz, Ahvaz, Iran. Initially, all rats were acclimatized to new environmental conditions for 1 week before the beginning of the experiment. Animals were randomly divided into 5 equal groups (n=5 in each group) as follow: group I: rats were fed with standard diet, group II: HFD-STZ-induced diabetic rats, group III: HFD-STZ-induced diabetic rats received *A. esculentus* (200 mg/kg) (13). The *A. esculentus* powder was mixed with normal diet and administrated orally. Group IV: HFD-STZ-induced diabetic rats received metformin (200 mg/ kg) (14), group V: rats received normal diet and *A. esculentus* (200 mg/kg) (13). Groups II, III and IV were fed with HFD for 4 weeks, whereas groups I and V consumed normal diet during the same period. 

The fat content of HFD was adjusted to 60% by addition of beef tallow into normal diets (15). Normal diet contained pellet chow of standard composition containing all the recommended macro and micro elements (56% carbohydrate, 18.5% protein, 8% fat, 12% fiber and adequate levels of minerals and vitamins). After 4 weeks of feeding the animals with HFD, rats were treated with a single dose of STZ (35 mg/ kg, i.v) (Sigma, Germany) that was prepared in citrate buffer 0.1 M (pH=4) (16). Five days after STZ treatment, glucose was measured by a hand-held glucometer (Medisign, China) and diabetes induction was confirmed if blood sugar was >250 mg/dl. The day after diabetes confirmation was considered as day 0 of treatment. After confirmation of diabetes induction, animals of groups III and V were orally treated with *A. esculentus* powder at the dose of 200 mg/kg body weight for 30 days and group IV was treated with oral metformin (200 mg/kg) for the same period. Animals in groups II, III and IV received a high-calorie diet throughout the experiment, while rats in groups I and V had access to standard diet during the experiment. The body weight and fasting blood sugar (FBS) were determined every week during the experimental period. 

### Serum biochemical assays

After overnight (12 hour) fasting, the rats were anesthetized using ketamine 100 mg/kg and xylazine 10 mg/kg on day 30 after initiation of the treatment. Heart blood samples were collected, and sera were separated and stored at -20°C for future use. Serum glucose was measured using a commercial kit (Pishtazteb, Iran) according to the manufacturer protocol. Insulin concentration was measured using a species specific ELISA kit (Koma Biotech Inc., South Korea) in a multiplate ELISA reader (BioTek, CA, USA) based on the protocol recommended by the manufacturer. Lipid profile including triglycerides (TG), total cholesterol (TC) and high density lipoprotein-cholesterol (HDL-c), was evaluated by enzymatic assay kits (Pars Azmoon, Iran). The serum low-density lipoprotein-cholesterol (LDL-c) and very-low-density lipoprotein-cholesterol (VLDL-c) concentrations were calculated using the Friedewald formula: LDL-c=TC-(HDLc+ VLDL-c) and VLDL-c=TG/5 (17). 

### Homeostasis Model Assessment of Basal Insulin Resistance Estimation 

For homeostasis model assessment of basal insulin resistance (HOMA-IR) the following equation was used: HOMA-IR=Fasting insulin level (µU/ml)×fasting blood glucose (mmol/l)/22.5. Lower HOMA-IR values demonstrated greater insulin sensitivity, and higher HOMA-IR values demonstrated lower insulin sensitivity (insulin resistance) (18). 

### RNA isolation and cDNA synthesis

At the end of the experiment, animals were scarified and pancreas tissues were immediately collected and frozen at -70°C. Total RNA was isolated using RNX TM reagent according to the manufacturer’s procedure (CinnaGen, Iran). Concentration of extracted RNA was calculated at a wavelength of 260 nm using nano drop spectrophotometry (Eppendorf, Germany). To detect the purity of RNA, its optical density (OD) ratio at 260/280 nm was determined and samples with a ratio of >1.8 were used for cDNA synthesis. Reverse transcription was carried out using the Rocket Script RT PreMix kit using 1 µg of RNA and random hexamer, based on manufacturer’s protocol (Bioneer Corporation, South Korea). Reverse transcription was carried out at 42°C for 90 minutes followed by incubation at 80°C for 3 minutes. cDNAs were stored at -20°C until used in the real-time polymerase chain reaction (PCR). 

### Real-time quantitative real-time polymerase chain reaction

To evaluate the expression levels of *PPAR-γ* and *PPAR-α* in the pancreas, real-time PCR analysis was performed using qPCRTM Green Master Kit for SYBR Green I^®^ (Jena Biosciense, Germany) on a Lightcycler^®^ Detection System (Roche, USA). Relative expression level of *PPAR-γ* and *PPAR-α* transcripts were compared to rat *GAPDH* as the housekeeping gene. Reactions were performed using 12.5 µl mixtures containing 6.25 µl qPCR^TM^ Green Master Kit for SYBR Green I^®^ (Jena Biosciense, Germany), 0.25 µl of each primer (200 nM), 3 µl cDNA (100 ng), and 2.25 µl nuclease-free water. The PCR protocol consisted of a 5-minute denaturation at 94°C followed by 45 cycles at 94oC for 15 seconds, and at 60°C for 30 seconds. Reactions were performed in triplicate. Two separate reactions without cDNA or with RNA were performed in parallel as controls. Relative quantification was performed according to the comparative 2^-ΔΔCt^ method using Lightcycler 96^®^ software. Validation of assay, in order to check that primer for target genes and GAPDH had similar amplification efficiencies, was performed as described previously. All qPCR analyses were performed according to The Minimum Information for Publication of Quantitative Real-Time PCR Experiments (MIQE) guideline (19).

### Histological study

Rat pancreas samples were taken from all groups for histological studies. The samples were taken from gastric and splenic regions of pancreas. They were fixed in 10% buffered formalin immediately upon removal. Next, samples were dehydrated by passing through a graded series of ethanol and embedded in paraffin blocks. Then, 5-6-µm sections were prepared using routine paraffin embedding methods and the sections were stained by H&E. To clarify the effect of diabetes induction on ß-cells mass, the aldehyde fuchsin staining was performed on paraffin embedded sections of pancreas samples (20). Histological parameters including the number of ß cells, size of islets, cytoplasmic vacuolization and tonality of insulin granules within the cytoplasm of ß-cells were evaluated in histological analysis. 

For an exact estimation of the number of ß-cells, in each slide, islets were divided into two categories of large islets (A) and small islets (B) according to their approximate diameter. The larger islets (>200 µm) (21) had open spaces and higher numbers of ß-cells per islet and the small islets (<200 µm) had little space and fewer ß-cells per islet.

In order to count ß-cells, 30 slides from the pancreas of each group were randomly selected for histometrical analysis. Then, 10 microscopic fields of equal size were screened. The number of ß-cells in large and small islets, separately, was assessed by counting all nuclei of purple-violet stained cells inside one islet in the field (22). Approximately 10 islets were examined on each section. For each animal, 5 sections were counted and a total number of 250 large and small islets of each group were counted. All measurements were performedunder light microscope using Dino Lite lens (with Dino capture software, FDP2, Taiwan) at ×40 magnification.

### Statistical analysis

Data analyses were done using SPSS 16.0 software package (SPSS Inc., Chicago, IL, USA). The data are reported as mean ± SD. One way analysis of variance (ANOVA) followed by Tukey’s post-test for multiple comparisons were used to assess the variations in means among the groups. The level of significance for all tests was set at P<0.05.

## Results

### Proximate composition, and flavonoid and phenolic contents of *A. esculentus*

The results of the present study showed that concentrations of the total phenolic and flavonoid compounds in the *A. esculentus* extract were 141 mg gallic acid/g of dry extract and 147 mg quercetin/g of dry extract, respectively. The moisture of dried *A. esculentus* was 13.7%. Carbohydrates, proteins and ash contents of dried plant were 1.6, 8.4 and 0.63 g/100 g, respectively.

#### Effects of *A. esculentus* on serum diabetes markers

**Fig.1:**
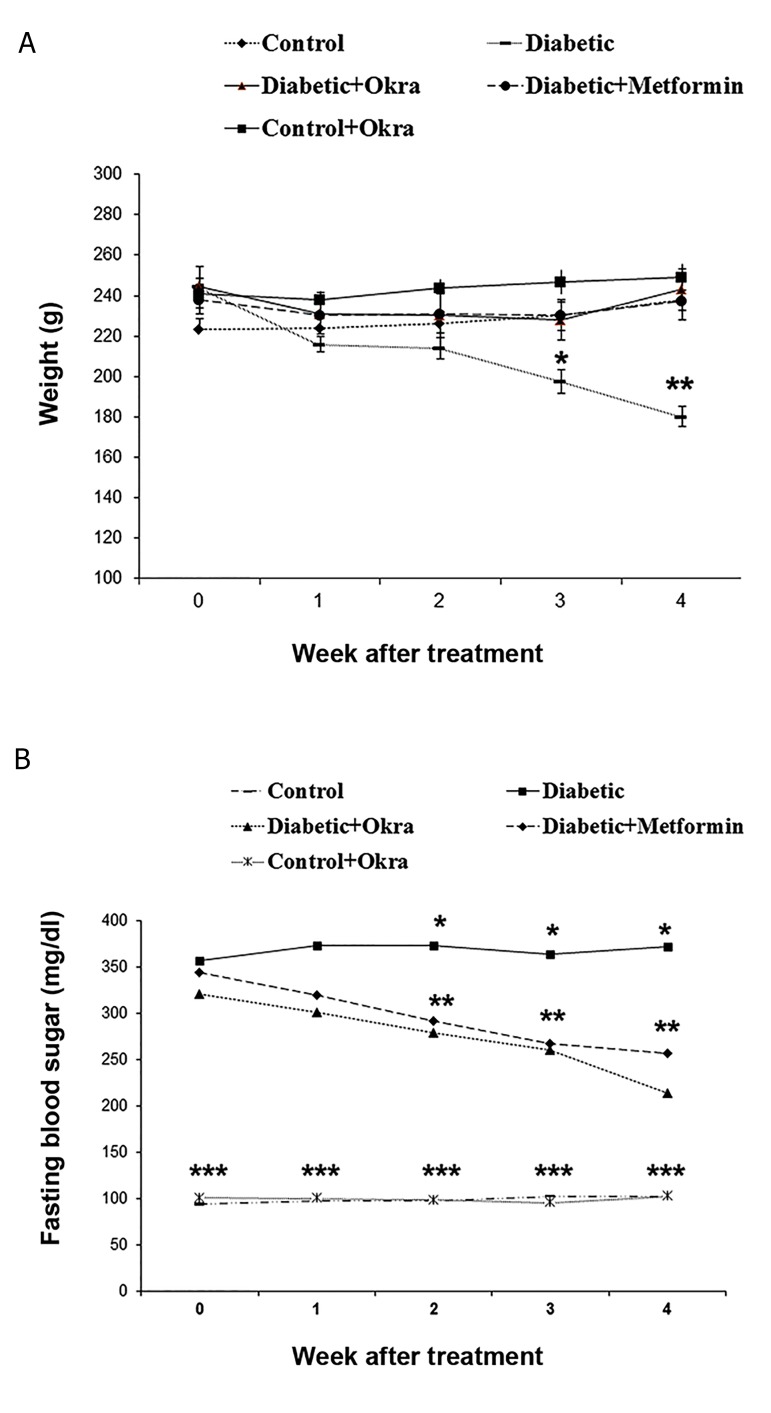
The serum glucose levels and body weight changes in different groups. Different numbers of * show significant difference (P<0.05). A and B. Are serum glucose level and body weight changes, respectively.

Serum glucose level was significantly increased in HFD-treated diabetic rats compared to the control group. Treatment of HFD-treated diabetic rats with *A. esculentus* for 30 days, significantly reduced blood glucose level compared to the untreated diabetic rats (P<0.05). The reduction of blood glucose level was not significantly different between HFD-treated diabetic rats treated with *A. esculentus* and those treated with metformin. Also, A.esculentus had no significant effects on blood glucose level in control group (Fig .1A). 

Fasting serum insulin level and HOMA-IR in HFD- treated diabetic rats following treatment with *A. esculentus* are shown in Table 1. HOMA-IR that had higher level in HFD-treated diabetic rats compared to control rats (P<0.05), was significantly decreased after administration of *A. esculentus* and metformin (P<0.05). HOMA-IR was decreased in control rats which received *A. esculentus*. Conversely, serum insulin level was decreased in HFD- treated diabetic rats compared to normal ones, while it was increased following treatment with *A. esculentus* for 30 days. Metformin had no obvious effects on serum insulin level in HFD-treated diabetic rats (P>0.05). *A. esculentus* had no significant effect on insulin level of healthy rats which received *A. esculentus* (Table 1). 

#### Effects of *A. esculentus* on diabetic rats’ body weight

At the end of 5-week HFD feeding, the mean weight gain of the HFD-treated diabetic rats was not significantly changed, while body weight was significantly decreased in HFD-treated group after STZ administration as compared to control group. HFD-treated diabetic rats which received A.esculentus and metformin for 30 days showed a significant increase in body weight as compared to HFD-treated diabetic rats (P<0.05). The effect of metformin on improvement of weight loss of HFD-treated diabetic rats was similar to thatof *A. esculentus*. No obvious body weight changes were observed in control rats treated with *A. esculentus* (Fig .1B).

**Table 1:** Serum lipid profiles, insulin levels and HOMA-IR in in different groups


Factor	TG	TC	LDL-c	VLDL-c	HDL-c	Insulin	HOMA-IR
Group							

**Control**	46.99 ± 2.32^a^	117.90 ± 9.05^a^	79.43 ± 9.54^a^	9.39 ± 0.46^a^	28.94 ± 1.41^a^	87.08 ± 9.2^a^	10.56 ± 3.32^a^
**Diabetic**	93.22 ± 25.12^b^	140.13 ± 19.5^b^	91.15 ± 18.17^b^	16.91 ± 5.02^b^	22.03 ± 3.04^b^	47.9 ± 3.2^b^	21.77 ± 1.45^b^
**Diabetic received *A.esculentus***	52.83 ± 1.92^a^	98.89 ± 5.02^a^	63.79 ± 4.87^a^	12.16 ± 1.63^a^^b^	22.93 ± 1.01^b^	62.06 ± 3.96^c^	17.8 ± 4.47^c^
**Diabetic received metformin **	57.03 ± 3.96^a^	97.60 ± 2.32^a^	65.34 ± 8.48^a^	11.40 ± 0.79^a^^b^	22.90 ± 1.15^b^	51.05 ± 6.09^b^	15.86 ± 4.82^c^
**Control received *A. esculentus***	53.85 ± 4.54^a^	100.37 ± 2.46^a^	72.20 ± 6.70^a^	10.77 ± 0.90^a^	25.06 ± 0.34^b^	65.83 ± 9.48^c^	8.62 ± 0.54^d^


Values are mean ± SD, n=5 animals per group. Different letters in each column denote significant differences (P<0.05). TG; Triglyceride, TC; Total cholesterol, LDL-c; Low density lipoprotein-cholesterol, HDL-c; High density lipoprotein-cholesterol, VLDL-c; Very-low-density lipoprotein-cholesterol, and HOMA-IR; Homeostasis model assessment of basal insulin resistance.

#### Effect of *A. esculentus* on serum lipid profile of diabetic rats

Lipid profile including TG, cholesterol, HDL-C, LDL-C, and VLDL-C, of treated and untreated diabetic rats are presented in Table 1. Diabetic rats showed significantlyhigher levels of cholesterol, TG, and VLDL-C compared tothe non-diabetic group. Serum LDL-C levels in diabetic ratswere similar to those of control animal (P>0.05). In diabeticrats treated with *A. esculentus* and metformin for 30 days, serum TG and cholesterol levels were significantly reduced, while LDL-C and VLDL-C showed no significant changes(P>0.05). Serum HDL-C level was significantly decreased(P<0.05) in diabetic rats, while it remained unchanged inanimals treated with *A. esculentus* and metformin. Treatment of healthy rats with *A. esculentus* had no significant effect on serum lipids profile (P>0.05) (Table 1). 

#### Effect of *A. esculentus* on pancreatic expression of *PPAR-γ* and *PPAR-α*

**Fig.2:**
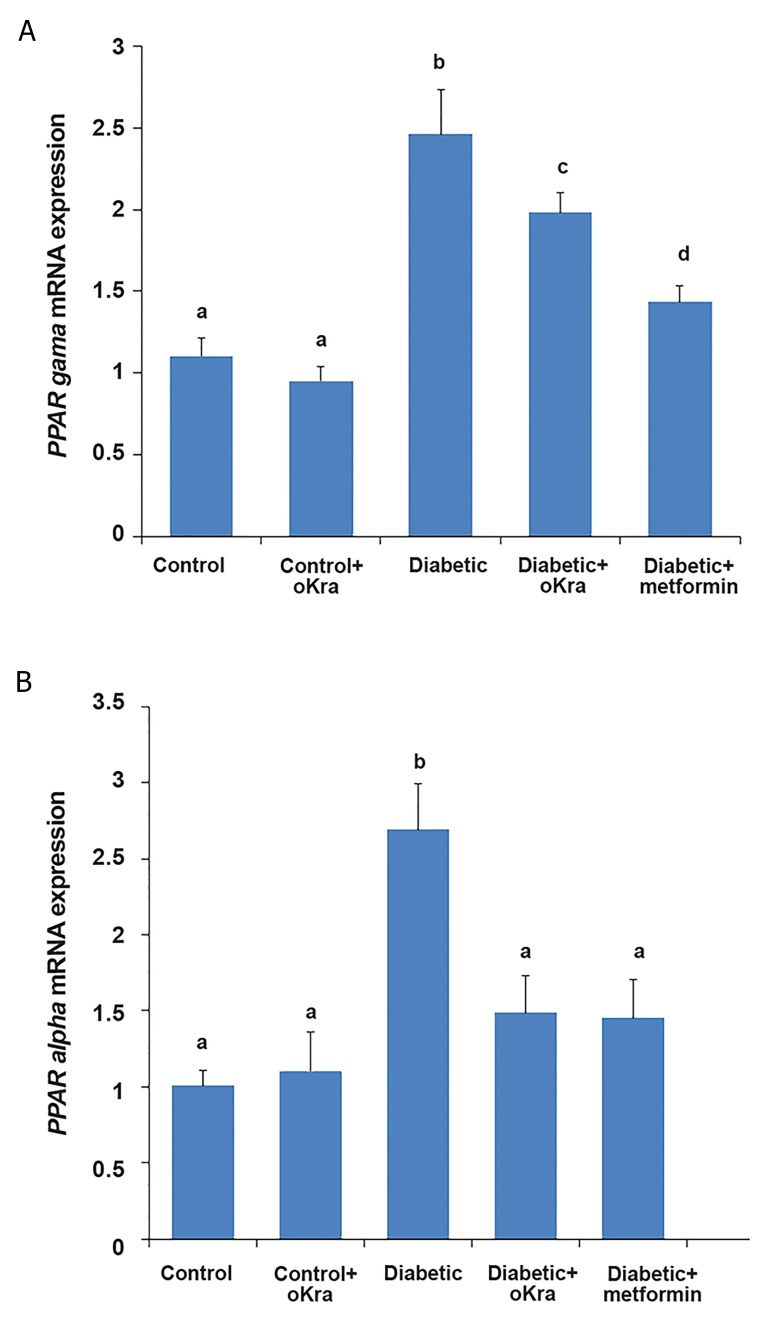
The *PPAR-γ* and *PPAR-α* mRNA gene expression in the pancreas in different groups. Data were presented as mean ± SD. Different letters denote significant differences (P<0.05). A and B. Are *PPAR-γ* and *PPAR-α* mRNA gene expression in the pancreas, respectively.

The expression levels of *PPAR-γ* and *PPAR-α* were significantly increased in the pancreas of HFD/STZtreated diabetic rats compared to the control rats (P<0.05). Treatment of diabetic animals with *A. esculentus* or metformin resulted in down-regulation of *PPAR-γ* and *PPAR-α* in the pancreas (P<0.05, Fig .2A, B). 

*PPAR-α* mRNA expression level in rats treated with A. esculentus and metformin was the same as that of control rats (Fig .2B). There were no significant changes in mRNA expression of pancreatic *PPAR-γ* and *PPAR-α* genes in healthy rats treated with *A. esculentus* (P>0.05, Fig .2A, B).

#### Effect of Okra powder on histological changes of pancreas

**Fig.3:**
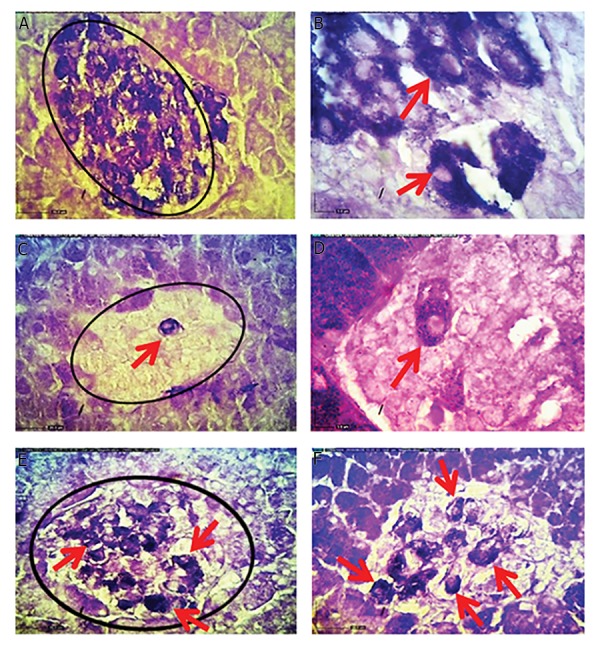
Histological changes of pancreatic islets in different experimental groups (H&E staining, ×40). A. Normal control rats, B. Normal control rats that received okra had normal pancreatic islets and ß-cells pancreatic composition, C, D. Pancreas of high-fat diet (HFD)-treated diabetic rats showed vacuolization (arrows), as well as reduction of islets size and ß-cells numbers, E. Pancreas of okra, and F. Metformin-treated diabetic HFD rats showed increased pancreatic islets size and ß -cells number (arrows), and decreased vacuolization.

**Fig.4:**
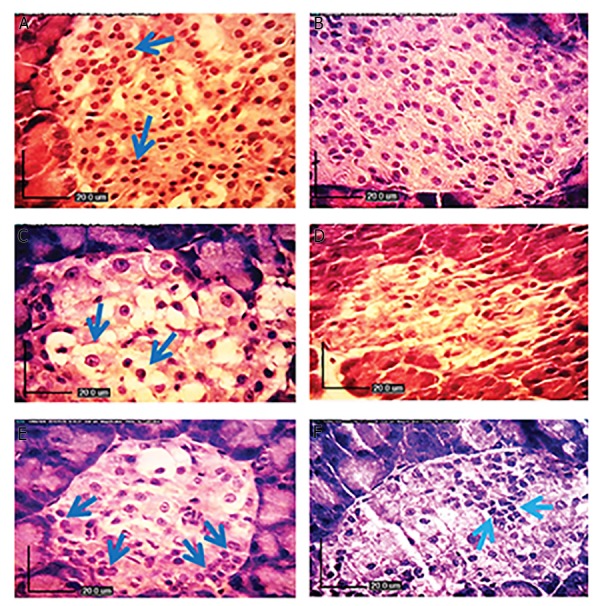
Aldehyde fuchsin staining of pancreatic islets in different experimental groups. A. Normal control rats (×40), B. (×100) showing normal ß-cells in the islets of Langerhans as well as distinct insulin granules filling the entire islets of Langerhans that are strongly stained in deep purple violet. High-fat diet (HFD)-treated diabetic rats, C. (×40), D. (×100) showing few surviving ß-cells in the islets of Langerhans and deficiency of their cytoplasmic tonality compared to control rats. The decreases in the reaction and the number of ß-cells are considerable (arrows, ×40), E. diabetic HFD rats treated with metformin, and F. diabetic HFD rats treated with okra showing remarkable increases in ß-cells mass, with heavily stained insulin granules.

Histological examination of the pancreatic islet tissues of experimental rats are presented in Figures 3 and 4. As seen in Figure 3A, the pancreatic islets of normal animals showed normal architecture. In contrast, islets of HFD treated diabetic rats showed severe pancreatic disruption, and vacuolization, as well as reduced islets’ size and relatively decreased number of ß-cells (Fig .3C, D). The severity of the above-mentioned changes was reduced in rats treated with A. esculentus and metformin compared to the untreated diabetic rats (Fig .3E, F). 

The number of ß-cells was assessed by counting all nuclei of the purple-violet stained cells inside the large and small islets of pancreas. The numbers of ß-cells in both large and small islets were decreased significantly in the pancreas of HFD- treated diabetic rats compared to the control rats (P<0.05). There was a significant increase in the number of ß-cells in large pancreatic islets of *A. esculentus* and metformin-treated groups compared to the HFD-treated diabetic rats (P<0.05). The number of ß-cells in small pancreatic islets in HFD- treated diabetic rats was increased significantly (P<0.05) following treatment with metformin, while *A. esculentus* had no significant effect on ß-cell mass of small pancreatic islets in HFD-treated diabetic rats (P>0.05). Treatment of healthy rats with *A. esculentus* caused no significant changes in the number of ß-cells in both large and small islets (P>0.05, Table 2). 

The results of the aldehyde fuchsin staining are shown in Figure 4. The normal cells in the islets of Langerhans showed distinct granules that were strongly stained in purple (Fig .4A, B). Diabetic rats (Fig .4C, D) demonstrated significant reductions in ß-cells and few surviving ß-cells were observed in the islets of Langerhans (P<0.05). Analyses of the pancreas of rats treated with *A. esculentus* and metformin (Fig .4E, F) showed remarkable increases in ß-cell, as reflected by purple granules (P<0.05). 

**Table 2:** The number of β-cells in large and small islets in the pancreas in different groups


Factor	Large islets (A)	Small islets (B)
Group		

**Control**	264.91 ± 16.92^a^	44.5 ± 2.13^a^
**Diabetic **	119.43 ± 15.15^b^	22.08 ± 1.89^b^
**Diabetic received *A. esculentus***	172.6111 ± 14.91^c^	25.27 ± 0.7^b^^c^
**Diabetic received metformin **	192.29 ± 16.55^c^	30.41 ± 3.01^c^
**Control received *A. esculentus***	253.91 ± 13.078^a^	47.16 ± 2.11^a^


Values are presented as mean ± SD, n=5 animals per group. Different letters in each column denote significant differences (P<0.05).

### Discussion

T2D is associated with decreased pancreatic ß-cells mass and function. Recently, focus on plant research has increased all over the world and a large body of evidence has shown the beneficial effects of medicinal plants on pancreatic dysfunction in diabetic patients (23). Recent findings have shown that *A. esculentus* can attenuate metabolic disturbances and insulin resistance related to diabetes in experimental animals (18), but its impact on pancreas histology and *PPARs*-dependent regulation in diabetes has not been clarified. In the present study, the effect of administration of *A. esculentus* on insulin resistance markers, serum lipid profile, pancreas structure and pancreatic expression of *PPARs* genes was determined in HFD/STZ-induced diabetic rats. 

In the present study, HFD/STZ-induced diabetic rats displayed elevated fasting blood glucose and HOMA-IR, accompanied by decreased serum insulin levels confirming the induction of diabetes in these animals. *A. esculentus* administration decreased blood glucose levels and HOMAIR and improved insulin resistance in HFD/STZ-treated rats. In accordance with our findings, Ramachandran et al. (24) have reported anti-diabetic activity of *A. esculentus* in alloxan-induced diabetic rats. Moreover, Sabitha et al. (25) have reported antidiabetic and antihyperlipidemic potential of okra peel and seed powder in STZ-induced diabetic rats. It has been found that administration of peel and seed powder of okra to diabetic rats reduces blood glucose level and increases body weight as compared to diabetic control (13). Various mechanisms have been proposed for antidiabetic action of *A. esculentus*. High concentrations of fiber and polysaccharides in fruits of *A. esculentus* can stabilize blood sugar by curbing the rate at which sugar is absorbed from intestinal tract (26). Khatun et al. (27) have also shown that water-soluble fraction of *A. esculentus* reduced the absorption of glucose from the intestine. 

Previous studies have shown that the extract of *A. esculentus* contains quercetin and its analogues. Recently, it has been found that quercetin decreases blood glucose levels in HFD/STZ-induced diabetic rats (18). Furthermore, quercetin and its analogues ameliorate insulin resistance in diabetic mice (28). Therefore, the phytoconstituents of okra might be responsible for antidiabetic property of this plant in diabetic rats. 

One major finding of our study was contrasting effects of *A. esculentus* on serum insulin level in diabetic and control animals. In accordance with previous works, our results showed that diabetes was associated with decreased pancreatic ß-cells mass and reduced serum insulin level. Therefore, we propose the hypothesis that *A. esculentus* ability to increase ß-cells mass was the key factor in restoration of insulin production and secretion in diabetic animals. ß-cells mass of control rats that received *A. esculentus*, had no significant difference with that of control, untreated rats, while serum insulin was obviously reduced in healthy *A. esculentus*-treated rats. Although the mechanism of insulin-lowering effect of *A. esculentus* in healthy animals is unknown, it may be indirectly related to increased ß-cells responsiveness to glucose as manifested by reduced HOMA-IR and insulin secretion. Thus, as insulin sensitivity increases in healthy *A. esculentus* treated rats, first-phase of insulin release may decrease proportionately to maintain normal glucose levels. However, further studies are needed to confirm this hypothesis. 

In the present study, diabetes was accompanied by an increase in TC, LDL-C, and TG and a reduction in HDL-C in HFD/STZ-induced diabetic rats. Treatment of HFD/STZ-induced diabetic rats with okra powder could profoundly improve lipid disturbances. Treatment of diabetic rats with *A. esculentus* notably reduced serum TG and TC levels while HDL-C levels remained unchanged. These results are in agreement with previous studies (29) that have shown that treatment with A. esculentus can reduce TC, total lipids and TG levels in rats fed with a HFD. Different mechanisms may underlie the improvement of lipid disturbances in diabetic rats after treatment with *A. esculentus*. Previous studies have shown that addition of *A. esculentus* to diet decreases the gene expression of SREBP1c and FAS (two key modulator of FA and cholesterol biosynthesis), which may finally reduce serum levels of TG and TC. Abundant dietary fibers present in *A. esculentus* are also capable of binding to bile acids consequently lowering TC through interfering with bile acids reabsorption (29). Roy et al. (30) reported that *A. esculentus* polysaccharides could decrease blood glucose levels in normal mice. 

*PPAR-α* and *PPAR-γ* are nuclear hormone receptors that maintain homeostasis of glucose in the pancreas. *PPARs* exhibit beneficial effects on metabolic abnormalities associated with T2D and also control the expression of various genes that are important for lipid and glucose metabolism (31). Studies have indicated strong correlations between *PPARs* activation and anti-diabetic effects of many herbal plants (23). More than 200 natural compounds, especially flavonoids, have been identified as agonist or antagonist of *PPAR-γ* receptors and may play roles in the prevention and treatment of metabolic disorders. In this regard, recent data has shown that A. esculentus can activate PPAR-γ in the liver of HFD- induced obese C57BL/6 mice. Because PPARs have insulin-sensitizing effects in peripheral tissues as well as the ability to sense blood glucose in pancreatic ß-cells, we attempted to evaluate whether okra may affect *PPARs* gene expression in the pancreas of diabetic rats. 

Quantitative real time PCR data showed that mRNA levels of *PPAR-γ* and *PPAR-α* were increased in HFD/ STZ-induced diabetic rats compared to control group rats. In accordance with our results, Zhou et al. (32) reported that, *PPAR-γ* expression is increased >5-fold in islets from Zucker diabetic fatty (ZDF) rats. In T2D, blood glucose and free FA levels are elevated, resulting in intracellular accumulation of TG within the pancreatic islets (33) and ß-cells secretory failure. Intracellular TG accumulation and overabundance of islet lipid, induce apoptosis in ß-cells by increasing free radicals formation. Over expression or activation of *PPAR-γ* and *PPAR-α*, up-regulates of key enzymes of mitochondrial and peroxisomal ß-oxidation and enhances FAs oxidation. It is reported that forced activation of *PPAR-γ* in the islets leads to stimulation of multiple metabolic pathways that help the disposal of FAs (34). Kakuma et al. (35) reported that long-term activation of *PPAR-γ* can reduce the lipid content of ZDF rat islets. Also, *PPAR-α* regulates the expression of genes involved in FAs and lipid metabolism (36). Studies performed in rodent models of insulin resistance indicated that *PPAR-α* activation by natural (FAs) or synthetic (fibrates) ligands, enhances insulin sensitivity by decreasing the lipid content of adipose and nonadipose tissues (7) or decreasing the endogenous glucose production (33). Based on these findings, we concluded that over-expression of *PPARs* in the pancreas of diabetic rats may be a compensatory mechanism for improvement of glucose sensitivity and ß-cells function. To confirm this hypothesis, previous studies have demonstrated that *PPARγ* over-expression can protect ß-cells function, morphology, and mass in rodent models of diabetes (34). 

Interestingly, *PPAR-γ* antagonists may ameliorate metabolic disorders such as obesity, insulin resistance and dyslipidemia, by inhibition of adipocyte differentiation. *PPAR-γ* antagonists, tanshinone IIAand ß-cryptoxanthine, have been reported to reduce body weight, blood glucose and serum TG in HFD-induced obese mice (37). Rieusset reported that dimethyl a-(dimethoxyphosphinyl)pchlorobenzyl phosphate (SR-202) as a selective synthetic inhibitor of *PPAR-γ* inhibits adipocyte differentiation and improves insulin sensitivity in diabetic ob/ob mice (38). Thus, *PPARγ* antagonists may be useful for the treatment of obesity-related insulin resistance. Our data showed that *PPAR-γ* and PPAR-α mRNA levels declined in diabetic animals treated with *A. esculentus* powder, confirming the PPAR antagonistic effect of *A. esculentus*. These results were in accordance with the findings of Fan et al. (18) that showed that okra consumption inhibits *PPAR-γ* and *PPAR-α* transcription in the liver of HFD-induced obese C57BL/6. 

Together, we suggest that okra may improve metabolic disorders related to diabetes through suppression of *PPARs* signaling. In addition to the above-described mechanisms, it seems that reduction of *PPAR-γ* and *PPAR-α* expression might be a consequence of improvement of hyperinsulinemia. In other words, increased *PPARs* expression during insulin resistance state, can improve insulin resistance, attenuate hyperlipidemia and increase overall glucose utilization, while improvement of insulin resistance after *A. esculentus* treatment results in down-regulation of *PPARs* in the pancreas. Based on these observations, it has been suggested that an increase in *PPARs* expression may induce a compensatory mechanism against progression of insulin resistance in obese patients (7, 18, 34). 

Additionally, our histological data showed that the size of islets and population of insulin-producing ß-cells were reduced in the pancreas of HFD/STZ-induced diabetic rats. Aldehyde fuchsin staining also demonstrated that *A. esculentus* powder could restore pancreatic ß-cells mass and reverse the ß-cells damage caused by HFD/STZ treatment. These results were in accordance with previous studies that demonstrated that hyperglycemia leads to a progressive decline in ß-cells function, the insufficiency of insulin secretion by the pancreatic ß -cells (39) and increased apoptosis in pancreatic islets (40). 

## Conclusion

Based on our data, okra could improve metabolic complications in an animal model of diabetes. Our results revealed that *A. esculentus* had beneficial effect on the pancreas of diabetic rats by restoration of ß-cell mass and modulation of PPAR-dependent pathways. The results of the present study provide new scientific evidence about therapeutic benefits of *A. esculentus* in diabetes. 
